# The Significance of Maximal Squat Strength for Neuromuscular Knee Control in Elite Handball Players: A Cross-Sectional Study

**DOI:** 10.3390/sports11120236

**Published:** 2023-11-27

**Authors:** Sofia Ryman Augustsson, Erik Gannby

**Affiliations:** 1 Department of Sport Science, Faculty of Social Sciences, Linnaeus University, 39231 Kalmar, Sweden; erikgannby@gmail.com; 2 Kry Health Center, 29535 Bromölla, Sweden

**Keywords:** knee valgus, relative strength, unilateral vertical drop jump, elite athletes

## Abstract

Both weak muscle strength and impaired neuromuscular control has previous been suggested as risk factors for future traumatic knee injury. However, data on the relationship between these two factors are scarce. Thus, the aim of this study was to investigate the relationship and influence of the one repetition maximum (1RM) barbell squat strength on dynamic knee valgus in elite female and male handball players. In this cross-sectional study 22 elite handball players (7 females) were included. A unilateral drop jump (VDJ) test was used for the assessment of frontal plane dynamic knee valgus. Players also performed a one repetition maximum (1RM) barbell squat test, expressed relative to bodyweight (r1RM), to assess maximal strength, which were dichotomized to analyze ‘weak’ versus ‘strong’ players according to median. Correlations were noted between r1RM in squat and knee valgus angle for both the non-dominant (r = −0.54; *p* = 0.009) and dominant leg (r = −0.46, *p* = 0.03). The odds of knee valgus were eight times higher, for the dominant leg, in the weak group compared to the strong group (*p* = 0.03) and 27 times higher, for the non-dominant leg (*p* = 0.002). The outcome of the present study suggests that maximum squat strength plays an important role when it comes to neuromuscular control of the knee, and that weak handball players are at higher risk of knee valgus compared to strong players during jumping activity.

## 1. Introduction

The anterior cruciate ligament (ACL) injury is the most common serious knee injury among handball players [1] and has been suggested to be higher in handball compared to other ball sports such as soccer, basketball, and volleyball players [2]. There are several factors that increase the risk of sustaining an ACL injury. For example, reduced maximum muscle strength of lower extremity [3,4] and poor neuromuscular control (e.g., knee abduction moment and imbalanced sequences of neuromuscular firing during dynamic actions) [5,6] have been described as risk factors. From a biomechanical standpoint, there is an increased prevalence of ACL injury when there is a combination of valgus collapse near full knee extension and Tibial rotation [7]. Thus, poor neuromuscular control, and especially increased knee valgus has been suggested to be a risk factor for ACL injury [5,8].

Neuromuscular control can be defined as the ability to produce controlled movements through coordinated muscle activity [9]. Regarding neuromuscular control of the knee, the knee joint position is often described as normal, varus, or valgus [10]. Knee valgus means that the angle between the reference points of the anterior superior iliac spine (ASIS), the midpoint of the patella, and the midpoint between the lateral and medial malleoli is less than 180° [11]. Knee valgus refers to the phenomenon when the distal femur moves medially towards the body’s midline while the distal tibia moves laterally away from the body’s midline [5]. Within handball medicine research, neuromuscular training is a well-known concept. According to several studies [12,13,14,15], neuromuscular training program can significantly reduce the risk of serious knee injuries and other lower extremity injuries among handball players.

Muscle strength is likely an important factor in maintaining good neuromuscular knee control, as the dynamic stability of the knee joint is largely dependent on the surrounding muscles [11]. According to Wilczyński et al. [11], reduced strength in the knee flexors and extensors, as well as the hip abductors, extensors, and external rotators, results in an increased risk of knee valgus during landing after jumps. In addition, previous research has indicated that weak lower extremity muscle strength can predict ACL injury in athletes and that high level of muscle strength is associated with a lower risk of traumatic injuries [3,4].

In summary, both weak muscle strength and impaired neuromuscular control appear to be relevant risk factors for the occurrence of traumatic knee injuries in sports. However, data on the relationship between these two factors seems to be relatively limited. Prior research have indicated that muscle strength seem to effect the degree of knee valgus angle [16,17,18], but no previous study seem to exist regarding the influence of maximum strength in barbell squat on knee valgus in elite athletes. It is well known that performance in barbell squat is transferable to many movements in sports and life such as jumping and sprinting [19,20,21] and that training squat also put stress on the neuromuscular system [22]. Hence, the squat is most likely the most popular exercise used by athletes to improve sport performance. The squat is also argued to be one of the most significant movements in the development of lower body strength, central nervous system adaptation and functional performance [23]. Hence, it is our belief that squat training not only increase muscle strength, it can also improve neuromuscular control of the knee such as reduced dynamic knee valgus. To our knowledge, there are no studies on the relationship between maximum barbell squat strength and dynamic knee valgus in elite athletes. The knowledge from the present study could help sport physical therapists, coaches and/or athletic trainers in the work of developing injury prevention strategies as well as rehabilitation protocol.

The aim of the present study was to investigate the relationship and influence of one repetition maximum (1RM) barbell squat strength on dynamic knee valgus in elite female and male handball players. We hypothesized that weaker squat strength would be associated with a higher degree of dynamic knee valgus.

## 2. Materials and Methods

### 2.1. Study Design and Participants

Data for cross-sectional study, adhering to the STROBE statement [24] were collected during 2023 where elite handball players were evaluated with 1RM test in barbell squat to measure lower extremity muscle strength and a vertical drop jump (VDJ), with unilateral landing, for biomechanical analysis of neuromuscular control of the knee (frontal plane dynamic knee valgus). In a first step, head coaches from representing 3 teams (one men’s team and two women’s team) were contacted and informed about the study. A total of 37 handball players (17 women) were asked to participate, out of which six declined to participate and nine were excluded. Thus, a total of 22 players, 15 men (representing top division) and seven women (three representing top division and four the second highest division), were included in the study (Table 1). Inclusion criteria were male and female handball players, aged 18–40 years, contracted by a handball club in Division 1 or higher, and familiar with performing the 1RM barbell squat test. Players with current knee, hip or back injury prior to testing and/or unavailable due to match play during the data collection period were excluded. Written information was given to each player and informed consent was obtained. All players achieved written information and written informed consent was obtained.

### 2.2. Procedure

Data collection took place at a gym, next to the handball arena, where the players usually perform their regular strength training. The players were divided into three groups for testing, and each group performed the test session at separate occasions to avoid disrupting their respective team’s schedule. The male players and three of the female players had no games seven days prior to the measurements, whereas four female players had played a match three days before the data collection. One of the authors (E.G.), a sport physical therapist, and the teams’ strength and conditioning coach, both experienced in instructing and supervising squats in a testing context for athletes, served as the test leaders during the 1RM barbell squat test. Before data collection, players were asked to indicate their dominant jumping leg (the leg they usually jump with in handball). Additionally, body mass (kg) was measured using a digital scale (Beurer SR BF2) at the gym whereas height was self-reported. All players performed the 1RM barbell squat test and the unilateral VDJ jumps in their regular training outfit (shorts or tight leggings and sports shoes). The test session began with a general warm-up on a stationary bike (Monark Ergomedic 828E) with self-selected resistance and speed for 5 min.

### 2.3. Measurements

#### 2.3.1. Muscle Strength Test

The 1RM barbell squat test is a measure of maximum muscular strength of the thigh and hip muscles (quadriceps, the gluteus muscles, hamstrings, and hip adductors [25]. The 1RM barbell squat test was performed according to previous protocol [26] and the relative 1RM, 1RM value expressed relative to body mass (r1RM), was used. The test began with 10 repetitions of the squat exercise with no extra load followed by 10 repetition of barbell squat with an Olympic 20-kg barbell (Eleiko, IWF weightlifting competition bar). All players were familiar with squats and started at a self-selected starting weight approximately 70% of their estimated 1RM. The barbell stand was individually adapted to each player’s height, so the barbell was placed at shoulder height. The players performed the squat by descending to a parallel squat position, approximately 110° of knee flexion, by bending her knees and hips until the greater trochanter of the femur reached the same horizontal plane as the superior border of the Patella. The weight lifted for each trial was increased, with free weight plates, by 2.5–10 kg (IWF weightlifting competition plate) until 1RM was reached and failure occurred. The test leader was responsible for the loading of every trial. 1RM was performed using 1-min resting periods between trials [26]. The number of unsuccessful trials before determining 1 RM in squat ranged between 4–6.

#### 2.3.2. Unilateral Vertical Drop Jump

Prior to each test, the test leader gave standardized instructions along with a visual demonstration of the test. The player was allowed one practice trial per side before initiating the testing. The VDJ test was performed according to previous protocol [27] with the player standing on a step-board, 30 cm high, on one leg and with their hands placed on the hips. The player dropped from the step-board, landing on one leg within an area marked with tape, then performed a maximal vertical jump upon landing. The marked area was used to ensure that the player landed within the range of the camera. The test was videotaped using a mobile camera (iPhone X), for later assessment of frontal plane dynamic peak knee valgus. To ensure that the whole movement was captured during testing, the camera was positioned 3 m in front of the player and in line with the waistline. The VDJ was performed on their dominant and non-dominant legs, respectively and peak knee valgus was assessed during the first landing, from first contact with the floor to extended knees. To facilitate analysis and to be able to measure knee valgus coach tape (SPORTDOC, Medical Blue) was used to mark landmarks on the SIAS, mid-patella, and mid-centered between the lateral and medial malleolus [11].

#### 2.3.3. Assessment of Dynamic Knee Valgus

**Figure 1:**
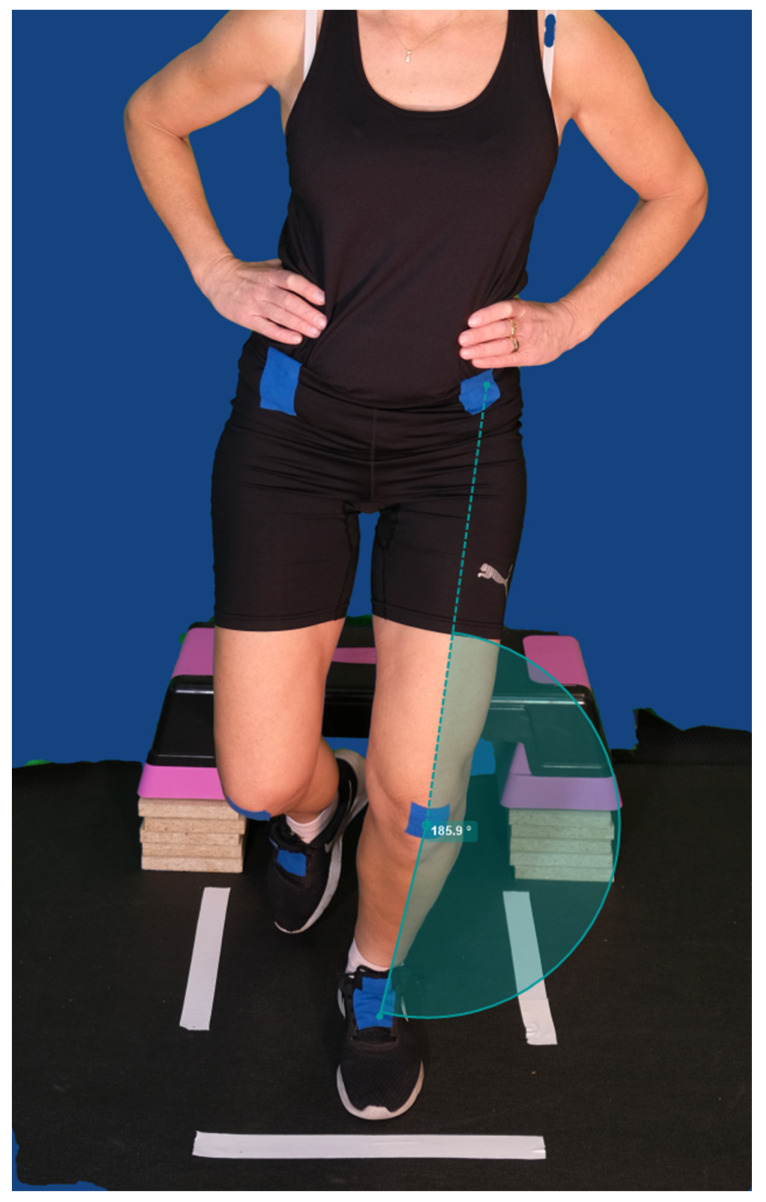
Landing with no knee valgus.

**Figure 2:**
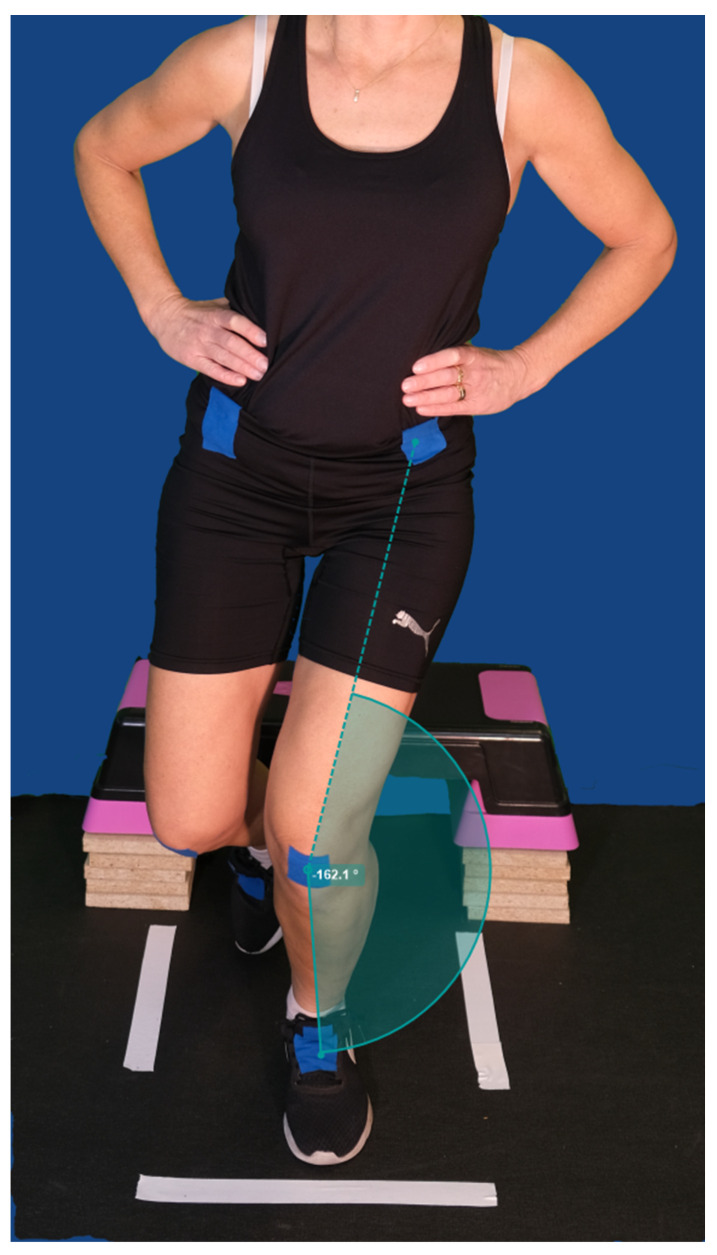
Landing with the presence of a valgus position.

One of the authors (E.G.), a trained physical therapist, observed and measured the degree of knee valgus from the video recording. Analysis and calculation of the valgus angle were performed using the software program Kinovea (version 0.9.5), which is a well-established software program designed to investigate and analyze kinematic parameters. The program has good reliability (ICC = 0.99) and validity for measuring angle degrees in sports testing contexts [28]. The taped reference marks served as the starting point for measuring. A straight line between the reference marks represented an angle of 180°, an angle greater than 180° indicated no knee valgus angle (Figure 1). A value below 180° indicated the presence of a valgus position (Figure 2). To calculate the degree of valgus angle, 180 (which corresponded to a straight line) was subtracted from the players’ measured value. Inter-rater reliability was assessed, in 10 players, by the two authors (EG and SRA) for the degree of knee valgus in VDJ with Cohen’s kappa [29] and showed excellent agreement (Cohen’s kappa value 0.89, *p* < 0.001), according to Landis and Koch [30]. Intra-rater reliability, analyzed on two separate occasions within 2 weeks, (assessed by the author EG) was calculated with intra-class correlation coefficient (ICC_2,1_), with the two-way random effect model (absolute agreement definition, 95% confidence intervals (CI)) and indicated excellent agreement (ICC_2,1_ value 0.99, *p* < 0.001).

### 2.4. Statistical Analysis

Statistics were calculated using IBM SPSS (IBM SPSS Statistics for Windows, Version 25.0. IBM, Armonk, NY, USA). To calculate if data (r1RM, VDJ) was normally distributed a Shapiro-Wilks test of normality was performed. The test showed that data for both tests in both male and female players were normally distributed (*p* ≥ 0.154), hence parametric statistics were used. Pearson’s correlation coefficient was used to examine any potential correlations between relative 1RM in barbell squat and the degree of valgus angle. Weak correlation was defined as r = 0–0.3, moderate correlation as r = 0.4–0.6, and strong correlation as r = 0.7–1.0 [31]. The strength variable (r1RM) was dichotomized to analyze ‘weak’ versus ‘strong’ players according to the median for female respectively male players [4]. Thus, the strong group consisted of three women and eight men whereas the weak group consisted of four women and seven men. To examine players with poor knee control and valgus compared to players with good knee control, without valgus, a valgus angle of ≥5° was chosen as the definition of valgus “yes,” and <5° as “no,” based on previous normative data [32]. The Independent-Samples T Test was used to compare means in knee valgus angle between strong and weak players, as well as valgus angle and r1RM strength values between sexes. The Pearson Chi-Square test was used to assess the association between r1RM strength (weak vs. strong) and the outcome of valgus (yes/no). Odds Ratio (OR), with 95% CI, was calculated to estimate the magnitude of muscle strength on the risk of knee valgus (“yes”). The significance level was set at *p* ≥ 0.05. Based on results from our previous study [16], investigating the influence of muscle strength on knee valgus, we hypothesized that there would be a 30% difference between weak and strong group in the number of players classified with knee valgus. The estimated number of participants required to achieve a power of 0.80 (α = 0.05) was 18. Therefore, this study was planned to recruit a minimum of 25 handball players with regard for potential dropouts.

## 3. Results

### 3.1. Correlations and the Influence of Maximal Squat Strength on Dynimic Knee Valgus

**Figure 3:**
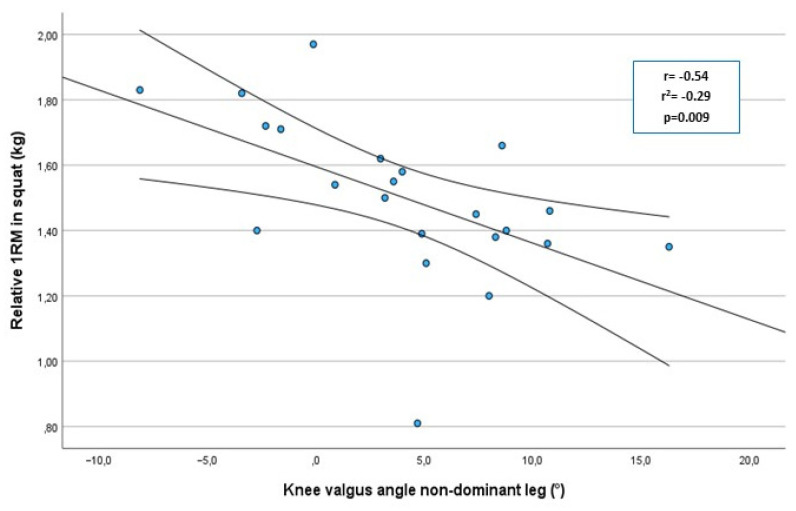
Correlation between knee valgus at the vertical jump drop jump test and the r1RM squat test for the non-dominant leg, in handball players (n = 22), with 95% confidence interval.

**Figure 4:**
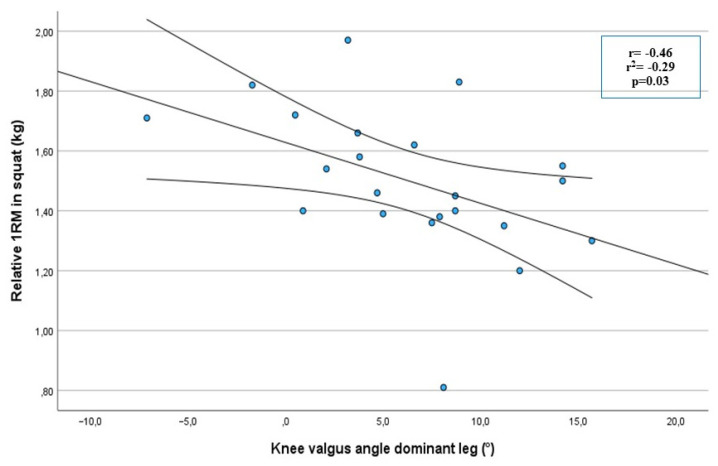
Correlation between knee valgus at the vertical jump drop jump test and the r1RM squat test for the non-dominant leg, in handball players (n = 22), with 95% confidence interval.

A significant moderate correlation was noted between the r1RM barbell squat and dynamic knee valgus angle for the non-dominant leg (r = −0.54; r^2^ = −0.29; *p =* 0.009) (Figure 3) as well as for the dominant leg, (r = −0.46; r^2^ = −0.21; *p =* 0.03) (Figure 4). A significant difference was noted between strong and weak players for the degree of knee valgus angle at the VDJ test for both the non-dominant leg (*p =* 0.003) and the dominant leg (*p =* 0.04) (Table 2). More weak players (8/11) were classified with knee valgus for the non-dominant leg compared to strong players (1/11) (*p =* 0.002). More weak individuals (9/11) were also classified with knee valgus compared to strong individuals (4/11) (*p =* 0.101) for the dominant leg. The odds of knee valgus were eight times higher, for the dominant leg, in the weak group compared to the strong group (OR 7.9; 95% CI 1.1 to 56; *p =* 0.03). The odds of knee valgus were 27 times higher, for the non-dominant leg, in the weak group compared to the strong group (OR 26.7; 95% CI 2.3 to 308; *p =* 0.002).

### 3.2. Differences between Men and Women

Men were significantly stronger in r1RM barbell squat compared to women (*p* = 0.003) (Table 3). A significantly higher knee valgus angle at the VDJ test was noted in the women compared to the men for the dominant leg (*p* = 0.007) as well as for the non-dominant leg (*p* = 0.048).

## 4. Discussion

The main results of this study were that weaker lower extremity muscle strength, measured with the 1RM barbell squat test, was associated with a higher degree of knee valgus angle during a VDJ test in elite handball players and that more weak players were classified with knee valgus compared to strong players. Thus, our hypothesis that weaker squat strength was associated with a higher degree of dynamic knee valgus, was confirmed.

### 4.1. Correlations and the Influence of Maximal Squat Strength on Dynamic Knee Valgus

To our knowledge, this is the first study investigating the influence of maximal squat strength on knee valgus in a VDJ test in elite athletes. In this study we found a moderate correlation between r1RM barbell squat strength and dynamic knee valgus angle for the non-dominant as well as for the dominant leg. The results of this study also indicate that maximal relative muscle strength measured with the 1RM barbell squat tests predicts dynamic knee valgus by 20 to 30% in elite handball players. Additionally, higher degrees of dynamic knee valgus angles were noted in weak players compared to strong players. More weak players were classified with knee valgus and the odds of knee valgus were eight times higher for the dominant leg and 27 times higher for the non-dominant leg in the weak group compared to the strong group. Thus, these results suggests that high levels of muscle strength are important for maintaining knee alignment during landing after jumping and reducing the risk of dynamic knee valgus. In line with our findings, prior research has reported that muscle strength seems to influence the degree of knee valgus angle [16,18]. For example, in one previous study it was noted that six weeks of strength and jump training, reduced degree of knee valgus during a VDJ was achieved [18]. In another study increased degree of knee valgus in VDJ was observed in weak individuals compared to strong individuals when isometric knee extension was examined [16]. In contrary, in another study, none of the muscle strength measures (quadriceps, hamstrings and hip) were associated with peak knee valgus angles [33]. One reason for the contradictive results could be derived from the measurements of muscle strength. Previous studies have either measured strength with an isokinetic dynamometer [16] or a handheld dynamometer [18,33]. In the present study we used a barbell squat test. The barbell squat test not only targets the lower extremity in a closed-kinetic chain position [34], but also challenges the core muscles [35] and the neuromuscular system extensively [22]. Prior research has demonstrated that core stability exercise alters neuromuscular function and decrease knee valgus angle during single-leg landing [36]. Thus, the activation of core muscle during the squat exercise may be one reason for the relation to knee valgus noted in the present study. Earlier research [4] also highlights the importance of squat strength in the work with preventing traumatic knee injury. The finding indicated that weaker lower extremity muscle strength seem to predict ACL injury, in youth female athlete [4]. Further, it has previously been recognized that there seems to be a relationship between higher knee valgus angle and increased risk of ACL injury [5,7,8]. Thus, interventions aimed at increasing strength and reducing degree of knee valgus are probably of relevance in preventing ACL injury.

In handball research, neuromuscular training is a well-known concept which has been shown to significantly reduce the risk of serious knee injuries [12,14,15]. Neuromuscular training aims to improve mechanisms responsible for dynamic control, joint stability and optimize neuromuscular transmission, a process that enables the central nervous system to control the movement of muscles [37,38]. However, it is also well-known that strength performance depends on the capacity of the nervous system to properly activate the muscles and that strength training causes adaptive changes within the nervous system such as increased motor unit recruitment, rate coding and improved motor unit synchronization [39,40,41]. Thus, strength training have been used to improve joint position sense, proprioception and muscle activation [42,43] and give athletes the ability to more fully activate prime movers in specific movements and to better coordinate the activation of relevant muscles [39].

In summary, the outcome of the present study suggests that maximal strength in squat is an important factor in maintaining knee alignment and decrease dynamic knee valgus. This supports the significance of the barbell squat as an exercise and useful tool in training program aiming for the improvement of neuromuscular knee control. However, the effect of such program must be further investigated.

### 4.2. Differences between Men and Women

Male players were significantly stronger in r1RM barbell squat compared to female (0.2 kg/kg body mass), which can be explained by various reasons. For example, women generally have lower muscle mass and less occurrence of type 2 muscle fibers [44] and are generally about 5–15% weaker than men in the lower extremity, measured in relative strength [44,45]. Another plausible explanation may be that the female player’s average age was 20 years, and the males was 25 years. This difference can be a factor of relevance as the men most probably have five years more experience in strength and conditioning training and for that reason have had time to develop their maximum strength to a greater extent than the women. However, we can only speculate about experience with strength training as no such data was collected in this study. Still, full development of muscle strength depends on full maturation of the nervous system which is reached usually by age 20 in women and between the ages 20 and 30 in men [44]. Hence, the female players in the present study have most probably reached full maturity and are at the same maturity level as the male players considering strength development.

A significantly higher degree of knee valgus angle was noted in the female players compared to the male players for both dominant leg and non-dominant leg (mean difference 6.5° and 4.8° respectively). This result is in line with previous research that has indicated that women land in 4.5–6.3° larger knee valgus than men in VDJ [46,47]. One reason for the higher degree of knee valgus noted in the female players in the present study may be that their relative strength was significantly lower than the male player’s. Previous research has noted that weak hip abductors can lead to increased knee valgus [17,48] and the hip abductors are a muscle group that is largely recruited during a squat [49]. Thus, one explanation for the women’s inferior relative strength could be that they were weaker than the men in the hip abductors and therefore demonstrated greater knee valgus. However, the nature of the barbell squat is multifactorial, involving the coordinated interaction of numerous muscle groups [22,34]. Aside from hip abductor muscle, the barbell squat generates quadriceps and hamstrings activity, as well as challenges the neuromuscular system of the prime movers to a great extent [22,34]. These are also factors that could be related to neuromuscular knee control such as maintaining knee alignment. Thus, a possible weakness of the squat test could be the difficulty to distinguish the relative influence of factors to failure in the squat test and, thus, to predict knee valgus. Moreover, it is important to point out that there may be other non-modifiable factors that can explain the difference noted between sexes regarding knee valgus, such as higher q-angle [50]. Still, unlike muscle strength, anatomical factors are difficult to influence.

### 4.3. Strengths and Limitations

This is, to our knowledge, the first study to provide data on the relationship and influence of muscle strength on knee valgus in elite handball players. Another strength of the present study is that the sample consisted of a homogeneous group of elite athletes. Another strength is the use of reliable and valid clinically applicable measurement for assessing maximal muscle strength [26,51] and knee valgus [28,52]. However, one limitation might be choice of cut-off value for poor knee control. To identify players with poor knee control, knee valgus was defined as having a knee valgus angle ≥ 5° which was based on previously published normative data suggesting a clinically meaningful angle [32]. The degree that defines valgus is relatively critical for the results of the study. However, to our knowledge, there is no current evidence on injury risk associated with a specific cut-off value for the degree of valgus. Rather, research indicates that, the higher the degree of valgus, the higher the risk of injury [5]. Thus, future studies are needed to determine the most appropriate cut-off value for knee valgus in relation to the risk of injury. Another limitation in the present study is the small sample of female players. For this reason, data for weak versus strong players is presented for the entire group and not broken down by sex. In addition, the players could be considered somewhat heterogeneous as they consisted of both women and men, ranging from 18 to 37 years old. However, they did not differ when it comes to experience of a specific sport and training, exercises testing and performing the 1RM barbell squat test. Thus, as stated above, the sample can be regarded as rather homogeneous. Yet, with respect to the small sample of female players, data regarding sex differences must be interpreted cautiously. The fact that higher degree of knee valgus angle and lower relative squat strength was noted in the female players compared to the male players suggests that future studies need to address this further with a larger sample divided by sex.

## 5. Conclusions

The outcome of the present study suggests that maximum squat strength plays an important role when it comes to neuromuscular control of the knee, and that weak handball players are at higher risk of knee valgus compared to strong players during jumping activity. This study underscores the significance of intervention studies evaluating whether enhancements in 1RM squat strength can be obtained and thereby improve neuromuscular knee control especially in female athletes who appear to be both weaker and have greater knee valgus than male athletes.

## Figures and Tables

**Table 1:** Players’ characteristics for female and male players (*n* = 22).

	Females (n = 7)	Males (n = 15)
	Mean (SD)	Mean (SD)
Age (years)	20 (3)	25 (5)
Weight (kg)	70 (6)	91 (12)
Height (cm)	171 (6)	188 (5)

**Table 2:** Dynamic knee valgus angle for the non-dominant and dominant leg in strong versus weak players respectively (n = 22).

	Strong Players (n = 11)	Weak Players (n = 11)	Differences
	Mean (SD)	Mean (SD)	Mean (SD)
Non-dominant leg (°)	1.6 (4)	6.7 (4) *	5.1 (6) *
Dominant leg (°)	4.2 (6)	8.5 (4)	3.8 (7) *

* = Significant difference between groups (*p* < 0.05).

**Table 3:** Relative 1RM barbell squat strength and knee valgus angle for dominant and non-dominant leg for women versus men (n = 22).

	Females (n = 7)	Males (n = 15)	Differences
	Mean (SD)	Mean (SD)	Mean (SD)
r1RM barbell squat (kg)	1.29 (0.2)	1.6 (0.2)	0.3 (2) *
Knee valgus non-dominant leg (°)	7.3° (4)	2.5° (5)	4.8 (7) *
Knee valgus dominant leg (°)	10.7° (3)	4.2° (5)	6.5 (8) *

* = Difference between groups (*p* < 0.05).

## Data Availability

The data presented in this study are available on request from the corresponding author.
